# Pufferfish (Tetraodon cutcutia) Sampled from a Freshwater River Serves as an Intermediate Reservoir of a Sucrose Nonfermenting Variant of Vibrio cholerae PS-4

**DOI:** 10.1128/spectrum.01221-21

**Published:** 2022-02-16

**Authors:** Lipika Das, Sushanta Deb, Eiji Arakawa, Shinji Yamasaki, Subrata K. Das

**Affiliations:** a Department of Biotechnology, Institute of Life Sciences, Nalco Square, Bhubaneswar, India; b Regional Center of Biotechnology, NCR Biotech Science Cluster, Faridabad, India; c Department of Bacteriology I, National Institute of Infectious Diseases, Tokyo, Japan; d Department of Veterinary Science, Graduate School of Life and Environmental Sciences, Osaka Prefecture University, Osaka, Japan; Howard University

**Keywords:** pufferfish, *Vibrio cholerae*, sucrose nonfermenting, serotyping, phylogenomic analysis, virulence genes

## Abstract

We describe the genomic characteristics of Vibrio cholerae strain PS-4 that is unable to ferment sucrose on a thiosulfate citrate bile salt sucrose (TCBS) agar medium. This bacterium was isolated from the skin mucus of a freshwater pufferfish. The genome of strain PS-4 was sequenced to understand the sucrose nonfermenting phenotype. The gene encoding the sucrose-specific phosphotransferase system IIB (sucR) was absent, resulting in the defective sucrose fermenting phenotype. In contrast, genes encoding the glucose-specific transport system IIB (ptsG) and fructose-specific transport system IIB (fruA) showed acid production while growing with respective sugars. The overall genome relatedness indices (OGRI), such as *in silico* DNA-DNA hybridization (*is*DDH), average nucleotide identity (ANI), and average amino acid identity (AAI), were above the threshold value, that is, 70% and 95 to 96%, respectively. Phylogenomic analysis based on genome-wide core genes and the nonrecombinant core genes showed that strain PS-4 clustered with Vibrio cholerae ATCC 14035^T^. Further, genes encoding cholera toxin (*ctx*), zonula occludens toxin (*zot*), accessory cholera enterotoxin (*ace*), toxin-coregulated pilus (*tcp*), and lipopolysaccharide biosynthesis (*rfb*) were absent. PS-4 showed hemolytic activity and reacted strongly to the R antibody. Therefore, the Vibrio cholerae from the pufferfish adds a new ecological niche of this bacterium.

**IMPORTANCE** Vibrio cholerae is native of aquatic environments. In general, V. cholerae ferments sucrose on thiosulfate citrate bile salt sucrose (TCBS) agar and produces yellow colonies. V. cholerae strain PS-4 described in this study is a sucrose nonfermenting variant associated with pufferfish skin and does not produce yellow colonies on TCBS agar. Genes encoding sucrose-specific phosphotransferase system IIB (sucR) were absent. The observed phenotype in the distinct metabolic pathway indicates niche-specific adaptive evolution for this bacterium. Our study suggests that the nonfermenting phenotype of V. cholerae strains on TCBS agar may not always be considered for species delineation.

## INTRODUCTION

Vibrios are ubiquitous and plentiful in aquatic environments, including estuaries, marine coastal waters, and sediments, and aquaculture practices worldwide (1, 2). Several studies have demonstrated that vibrios are associated with aquatic animals (3, 4). Due to rapid growth, salt tolerance, and biofilm-forming capacities, the genus *Vibrio* developed adaptive skills and the physiological flexibility to survive and flourish in the diverse oligotrophic environment (5). *Vibrio* is Gram negative under the class *Gammaproteobacteria*, is chemoorganotrophic and mesophilic, and usually has motile rods. Vibrios are either pathogenic (6) or nonpathogenic (7). Vibrio cholerae O1, O139, and non-O1/non-O139 are indigenous water-living microorganisms. To date, approximately 200 serogroups of V. cholerae are known. Only O1 and O139 serogroups are associated with cholerae epidemics and pandemics. However, V. cholerae reported from the aquatic environment acquired virulence genes or homologs with low or no pathogenicity (8). Thus, the emergence of pathogenic *Vibrio* strains in the environment is due to the exchange of genetic elements (9). Two virulence genes, cholera toxin (*ctx*) and toxin-coregulated pilus (*tcp*), found in O1 and O139 serotypes, are essential for the pathogenicity of V. cholerae (10). However, other non-O1/non-O139 strains are causing diarrhea due to the presence of secretion systems (type III secretion system [T3SS] and type VI secretion system [T6SS]) and other accessory toxins, such as zonula occludens toxin (Zot) (11). Additionally, they have other genes encoding hemolysin, helping to colonize the intestine (12). The absence of cholera enterotoxin in V. cholerae non-O1/non-O139 strains was also reported. Under favorable conditions, antigenic translation of V. cholerae non-O1/non-O139 to V. cholerae O1 has been demonstrated (13). Analysis of genome-wide single nucleotide polymorphisms (SNPs) is a widely accepted procedure for evaluating phylogenetic relations of V. cholerae pandemics (14). Additionally, genomic data can characterize endemic strains and evaluate V. cholerae transmission routes (15).

Previously, Vibrio mimicus was considered a biotype of V. cholerae. Subsequently, *V. mimicus* was recognized as a distinct species, as this organism was negative for sucrose fermentation (16). Few reports exist on sucrose nonfermenting or late-fermenting variants of Vibrio cholerae (17). Further, a phosphotransferase system (PTS) sucrose-specific IIB component mutation in Vibrio cholerae O1 strain IEC224 has exhibited a sucrose nonfermenting phenotype on thiosulfate citrate bile salt sucrose (TCBS) agar (18). Thus, whole-genome sequencing analysis may differentiate the sucrose fermentative and nonfermentative strains of V. cholerae for species delineation. Mutations in these metabolic pathways indicate different ecological adaptations of V. cholerae.

V. cholerae mostly inhabits aquatic environments. *Vibrio* spp. are often isolated from fish and fish products, and many species are pathogenic to different hosts. Recent evidence supports that fish can be the intermediate reservoirs and vectors of V. cholerae (19, 20). Indeed, fish and fish diet consumption cause cholera in different parts of the world (21, 22). We performed the culturable approach to isolate bacteria associated with skin mucus of freshwater pufferfish. Pufferfish belong to *Tetraodontiformes*, with regional names patkafish and fugu (23). Very little is known about freshwater pufferfish found in the rivers of the eastern part of India. Pufferfish produce tetrodotoxin (TTX) that leads to physiological disorders to human health, and several deaths were reported from Bangladesh (24, 25). The reason could be the presence of tetrodotoxin-producing bacteria (26). The presence of V. cholerae in the skin mucus of freshwater pufferfish is not known so far. Here, we described the biochemical characteristics, genomic analysis, and virulence properties of Vibrio cholerae strain PS-4 isolated from freshwater pufferfish.

## RESULTS AND DISCUSSION

### Isolation and identification of pufferfish skin-associated bacteria.

Fish mucosal surfaces are one of the most nutrient-rich sources of aquatic microorganisms. *Vibrio* was dominant in fish skin collected from estuaries, lakes, and rivers. Several species, such as V. cholerae, V. fischeri, V. vulnificus, V. furnissii, and *V. metoecus*, were identified as the dominant vibrios from fish skin (27). During this investigation, 26 bacterial strains were identified from the pufferfish skin. 16S rRNA gene sequence analysis identified these bacteria under 12 taxa belonging to *Gammaproteobacteria*, *Betaproteobacteria*, *Bacilli*, and *Flavobacteria*. The majority were assigned to the class *Gammaproteobacteria*. Bacteria identified from the mucus layer of pufferfish represent the genera Acinetobacter, *Shewanella*, *Bacillus*, *Aeromonas*, *Serratia*, *Moraxella*, *Delftia*, Staphylococcus, *Chryseobacterium*, *Exiguobacterium*, *Chromobacterium*, and *Vibrio*. All the strains were closely related to the respective bacterial taxa, with a 16S rRNA sequence similarity of more than 98% (Table S1 at https://figshare.com/articles/dataset/Supplementary_data-Table_S1_Figure_S1_pdf_txt-Supplementary_table_1/18865445). The mucosal skin surface and the associated microbiota protect the host against pathogens, contributing to host immune maturity (28), and serve as a natural niche for aquatic mucosal pathogen evolution (20). The diversity of *Vibrio* from clinical and environmental sources and its phylogenetic relationships are available. However, the presence of Vibrio cholerae species from the skin mucosal surfaces of pufferfish has not been reported so far (29). Like many other fish, no studies of the microbes associated with the skin mucosal surfaces of pufferfish and their distinction between potentially virulent versus nonvirulent strains are available. Thus, we used Vibrio cholerae strain PS-4 for detailed studies.

### Phenotype and serogroup of Vibrio cholerae strain PS-4.

**FIG 1:**
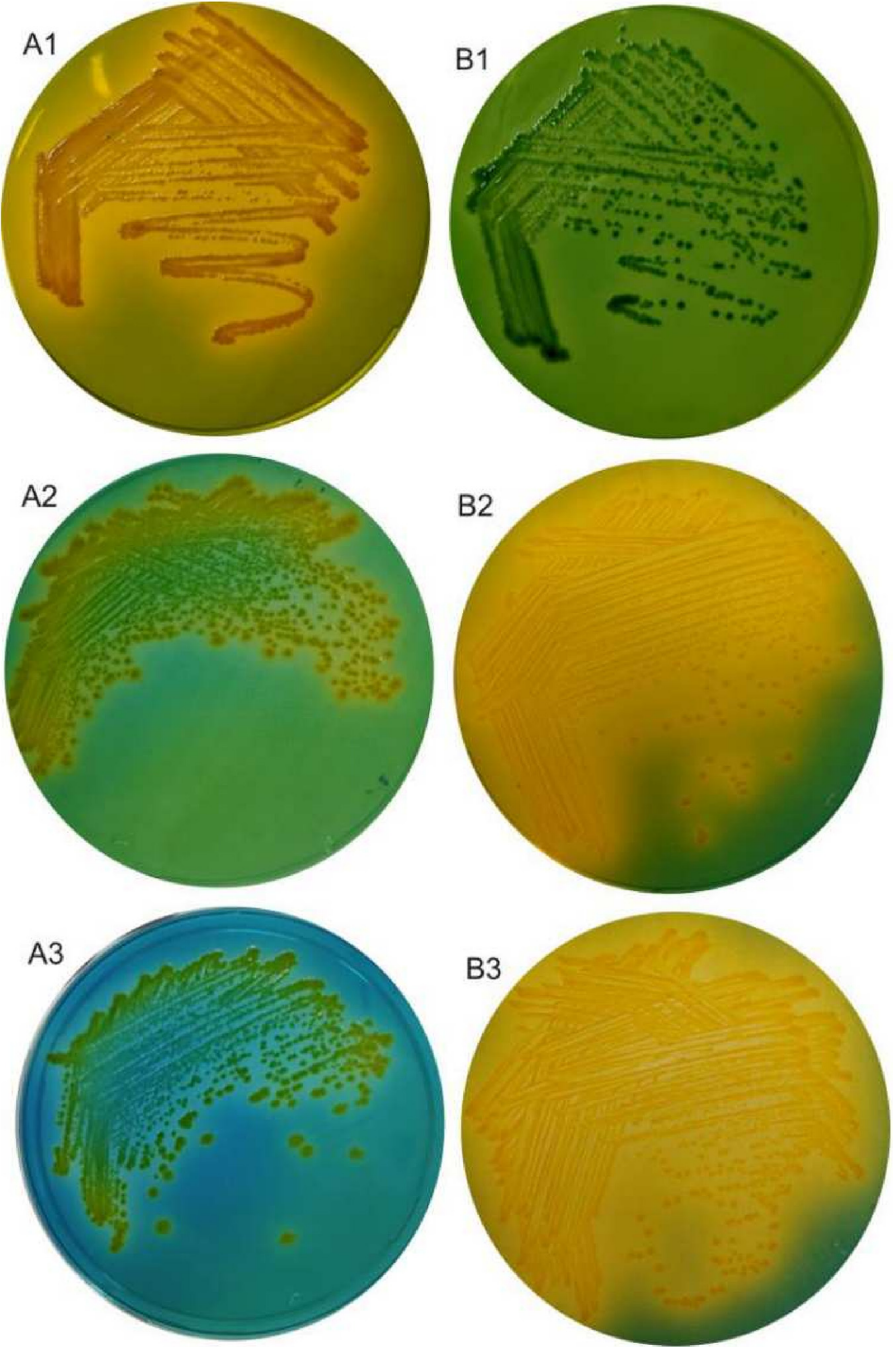
Growth responses of V. cholerae strain N16961 and V. cholerae strain PS-4 on TCBS (thiosulfate citrate bile salts sucrose agar) and Luria-Bertani agar supplemented with 0.5% glucose or 0.5% fructose and 2.0 mg/L bromothymol blue. V. cholerae N16961: A1, growth on TCBS; A2, growth on Luria-Bertani agar plus glucose; A3, growth on Luria-Bertani agar plus fructose. V. cholerae PS-4: B1, growth on TCBS; B2, growth on Luria-Bertani agar plus glucose; B3, growth on Luria-Bertani agar plus fructose.

The cells of strain PS-4 were Gram negative and positive for oxidase and catalase. PS-4 showed hemolytic activity on blood agar. Typically, V. cholerae produces yellow colonies on TCBS agar. In contrast, strain PS-4 was sucrose fermentation negative and had green colonies on this medium. In addition, PS-4 showed yellow colonies on Luria-Bertani agar medium supplemented with either glucose or fructose, similar to the Vibrio cholerae strain N16961 (Fig. 1). Genome analysis of the strain PS-4 revealed that the PTS system specific for sucrose IIB (sucR) was absent, accounting for the defective sucrose-fermenting phenotype. In contrast, genes encoding glucose-specific transport system IIB (ptsG) and fructose-specific transport system IIB (fruA) were present and showed acid production while growing in the presence of respective sugars. Our study based on biochemical characterization and genomic analysis suggested that the nonfermenting phenotype of Vibrio cholerae on TCBS agar may not always be considered for its species identification.

The serotyping result showed that strain PS-4 reacted strongly to the R (rough) antibody. Each antiserum was absorbed with the R antigen. Moreover, BLAST analysis of strain PS-4 scaffold sequences with the O antigen region of all O serogroups available in the NCBI database showed high homology with the part of the sequence of O127 antigen. Thus, the phenotype of the O antigen of strain PS-4 is R, but the genotype seems to be O127 (Table S1 at https://figshare.com/articles/dataset/Supplementary_data-Table_S1_Figure_S1_pdf_txt-Supplementary_table_1/18865445).

### Genomic features of Vibrio cholerae strain PS-4.

**FIG 2:**
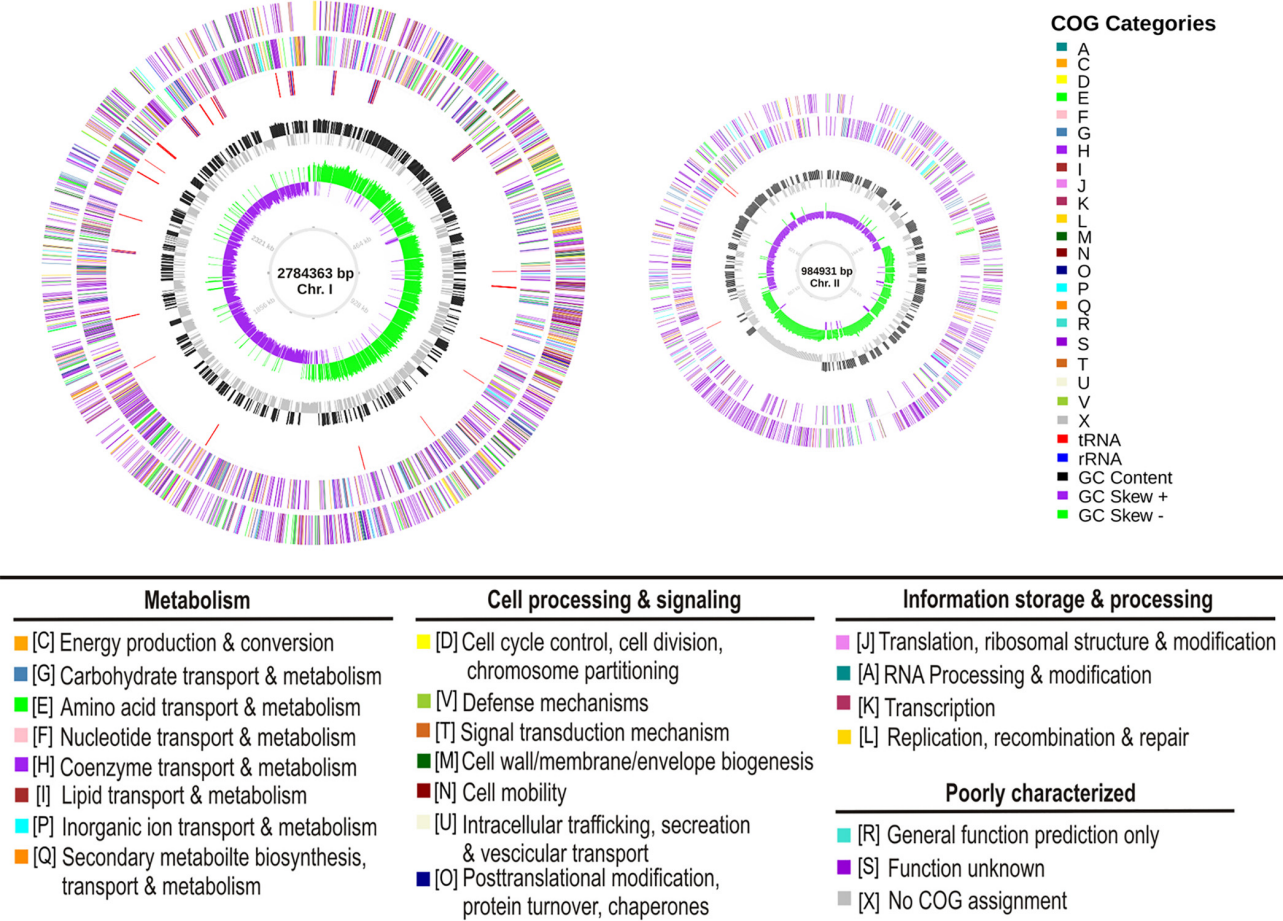
Circular graph of Vibrio cholerae strain PS-4 genome. Concentric outer to inner rings represent the protein-coding genes on the forward strand, protein-coding genes on the reverse strand, tRNA (red) and rRNA (blue) genes, GC content, GC skew, and scale marks of the genome. Protein-coding genes are color coded according to their COG categories.

The sequence of the V. cholerae strain PS-4 comprised two circular chromosomes, in which chromosome I contained 2,784,636 bp, while chromosome II contained 984,931 bp. The overall GC content was 47.61%. The genome consisted of 3,364 protein-coding sequences, of which 3,304 had a homologous function, 205 were predicted as hypothetical proteins, 31 were rRNA genes, and 104 were tRNA genes. The predicted open reading frames (ORFs) were further classified into clusters of orthologous genes (COGs) functional groups (Fig. 2).

### Genome-based analysis and phylogeny of Vibrio cholerae strain PS-4.

**FIG 3:**
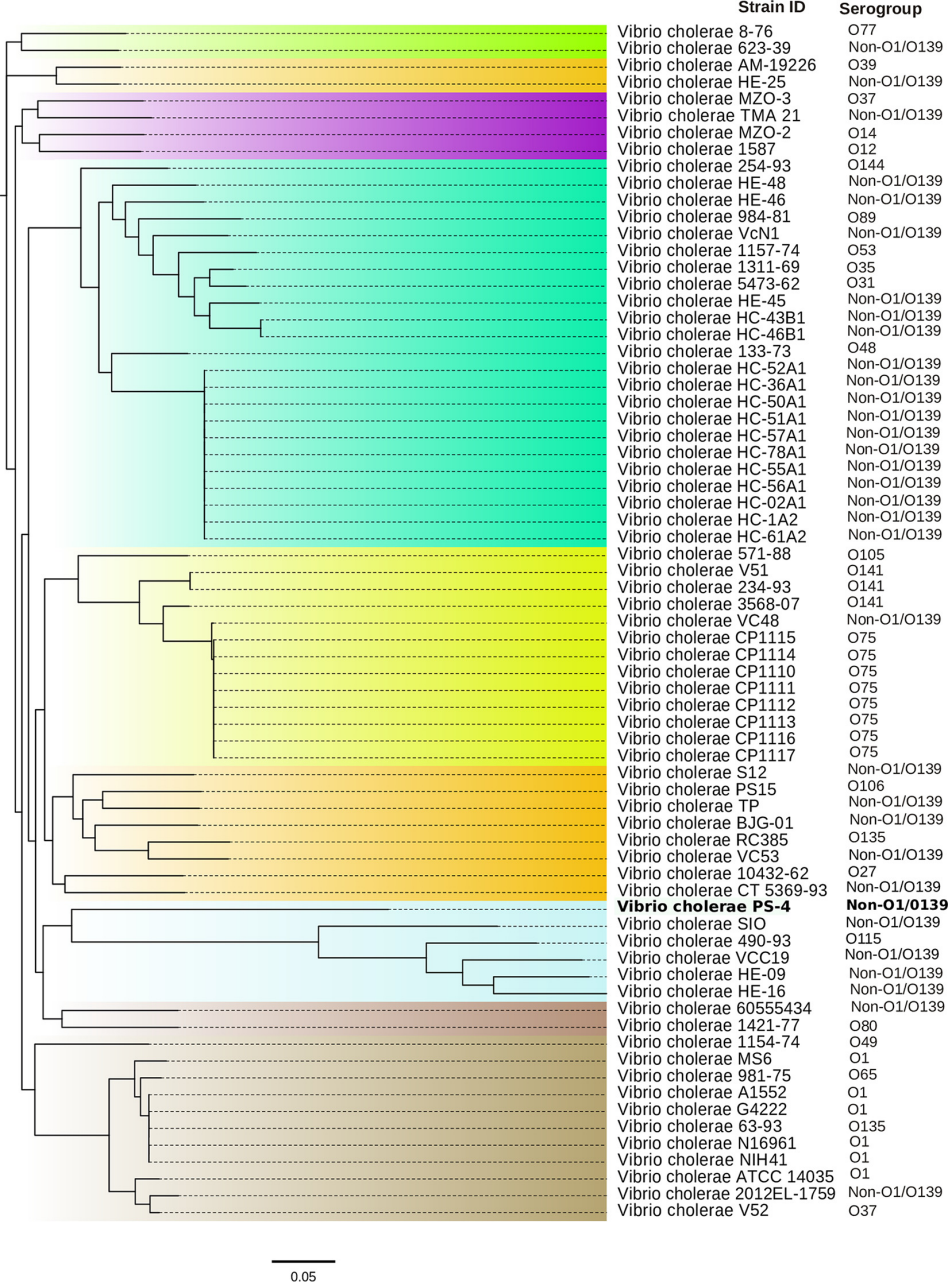
Maximum-likelihood phylogenetic tree based on genome-wide SNPs.

**FIG 4:**
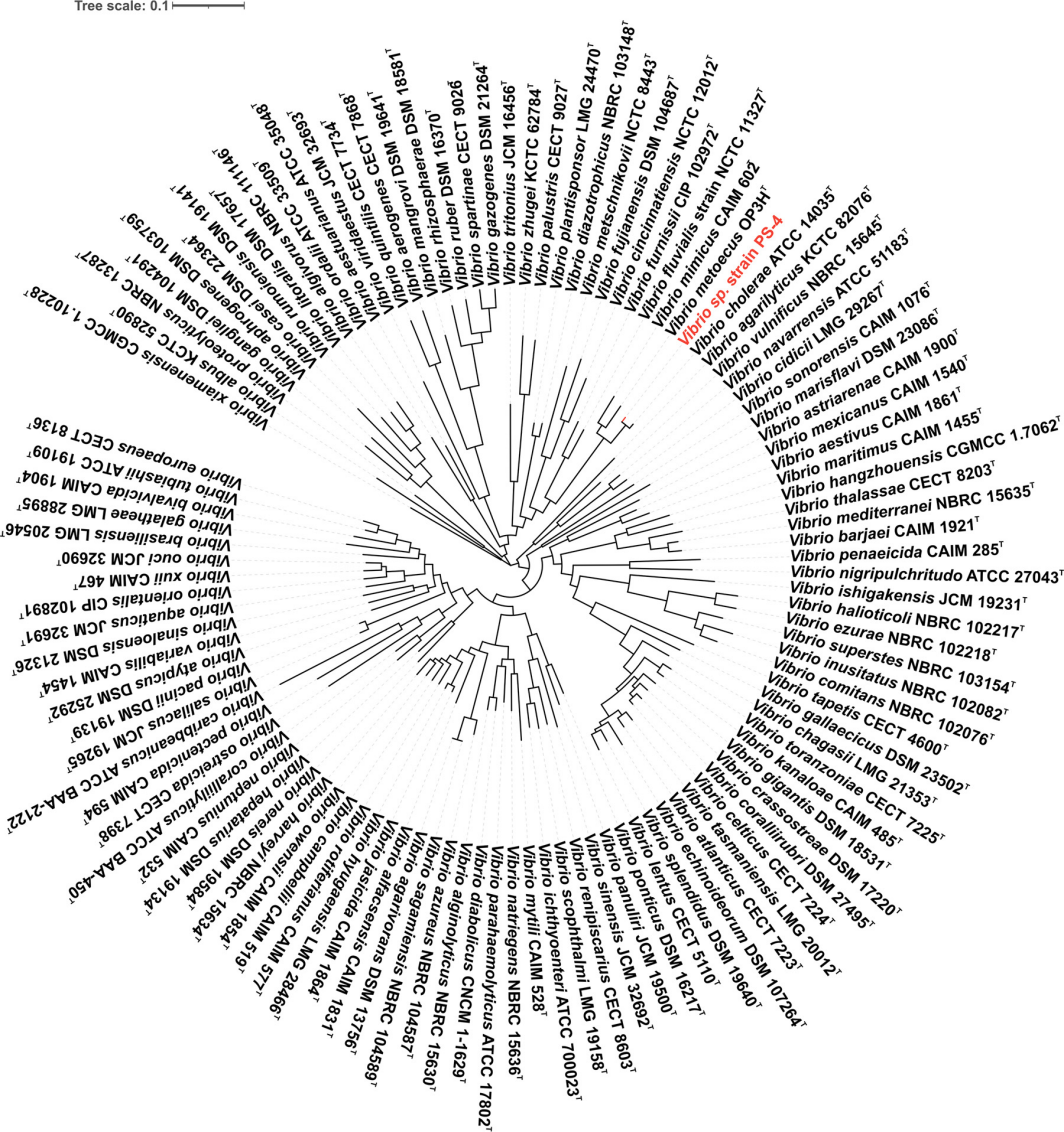
Core genome-based phylogenetic tree based on the alignment of core genes from 131 type strains of *Vibrio* of all species with correct validly published names. The phylogenetic position of strain PS-4 is highlighted in red.

Prokaryotic systematics is essential for the identification of microorganisms. Therefore, we evaluated the *in silico* DNA-DNA hybridization (*is*DDH) similarity, the average nucleotide identity (ANI), and average amino acid identity (AAI) values. Additionally, we conducted SNP-based phylogenetic analysis with the validly named type species to justify strain PS-4 belonging to V. cholerae. The ANI and AAI values between strain PS-4 and the type species of V. cholerae ATCC 14035 were higher than the threshold values (95 to 96%), justifying that both strains belong to the same species (30). Further, the *is*DDH similarity value was more than the cutoff value (70%) to define bacterial species (31). Thus, ANI, AAI, and *is*DDH data indicated that the strain PS-4 belongs to the same species of V. cholerae (Table 1). SNP-based phylogeny revealed that strain PS-4 clustered with non-O1/non-O139 V. cholerae strains (Fig. 3). The maximum-likelihood (ML) tree constructed on genome-wide core genes showed that strain PS-4, which clustered with V. cholerae ATCC 14035 (Fig. 4), should be considered now as belonging to V. cholerae. In addition, in the nonrecombinant core genome-based phylogenetic tree, strain PS-4 clustered with V. cholerae ATCC 14035 (Fig. S1 at https://figshare.com/articles/dataset/Supplementary_data-Table_S1_Figure_S1_pdf_txt-Supplementary_table_1/18865445), as found with the tree generated using core genomes (Fig. 4), indicating the robustness of tree topology.

**TABLE 1:** Comparison of the genomic characteristics with closely related species of *Vibrio*

Sl. No.	Strain	Accession no.^*a*^	Size (Mb)	16S rRNA similarity (%)	ANIb (%)	ANIu (%)	*is*DDH (%)	AAI (%)
1	Vibrio cholerae strain PS-4	CP077197 (Chr. I)	3.6	100	100	100	100	100
		CP077198 (Chr. II)						
2	Vibrio cholerae ATCC 14035^T^	NZ_JHXR00000000	4.0	99.93	96.4	96.5	70.2	97.3
3	Vibrio mimicus CAIM 602	NZ_AOMO00000000	4.3	99.59	87.2	86.4	31.6	91.8
4	Vibrio metoecus OP3H	JJMN00000000	3.9	99.27	85.9	85.3	29.8	92.3

a Chr. I, chromosome I; Chr. II, chromosome II.

### Virulence properties.

**FIG 5:**
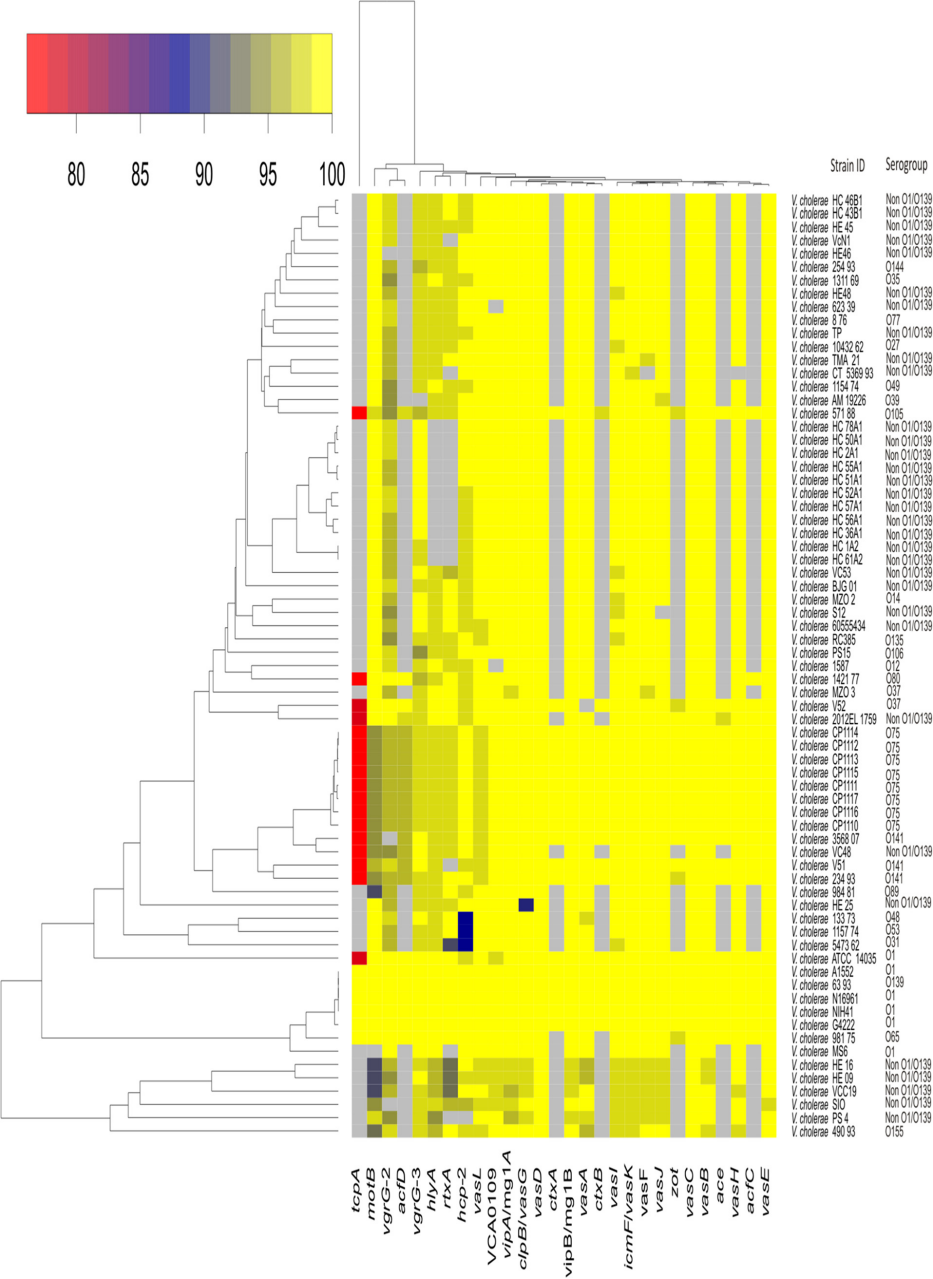
Conservation in nucleotide sequences of orthologous virulence genes in Vibrio cholerae strain PS-4 with reference strains. The top bar represents percent nucleotide sequence identity. Gray boxes show missing genes. The hierarchical clustering of the strains was based on average linkage method and Manhattan distance similarity metric.

Virulence genes identified in the genome of strain PS-4 are listed in Table 2. We compared 28 virulence-associated genes with 71 V. cholerae strains altogether. The individual gene sequences were compared with the reference toxigenic V. cholerae O1 El Tor strain N16961. Hierarchical clustering analysis illustrated that strain PS-4 shared maximum sequence similarity with nontoxigenic *Vibrio* isolates, *viz.*, HE-16, HE-09, VCC19, SIO, and 490 93, in a monophyletic clade (Fig. 5). Gene *hlyA*, responsible for the hemolytic activity, is occasionally reported from nontoxigenic non-O1/non-O139 serogroups (32, 33). The *hlyA* gene of strain PS-4 showed 97% sequence similarity to V. cholerae O1 El Tor strain N16961. However, other nontoxigenic strains (HE-16, HE-09, VCC19, SIO, and 490 93) of the same clade were showing sequence divergence (<98% nucleotide identity). The non-O1/non-O139 strains are mostly devoid of the *ctx*, *tcpA*, *zot*, accessory cholera enterotoxin (*ace*), and lipopolysaccharide biosynthesis (*rfb*) genes (10). Genome analysis revealed that *ctx*, *zot*, *ace*, *tcp*, and *rfb* were absent in PS-4; hence, this organism could be regarded as a non-O1/non-O139 serogroup. In V. cholerae, the type VI secretion system plays a critical role in delivering toxins into adjacent target cells and competing against other bacteria with toxins, disordering lipid membranes, actin cytoskeletons, and cell walls (34). The type VI secretion system consists of many virulence-associated secretion (*vas*) genes and *vgrG* effector protein (35). In this regard, the type VI secretion system of strain PS-4 encoded 15 genes. These genes showed sequence similarity of more than 94%, *viz.*, *vasL* (97.86%), *vipA* (97.83%), *vasG* (99.15%), *vasD* (99.37%), *vasA* (99.09%), *vasI* (97.71%), *vasK* (97.57%), *vasF* (97.02%), *vasJ* (98.29%), *vasC* (98.85%), *vasB* (98.42%), *vasH* (98.43%), *vasE* (98.05%), *vgrG*2 (97.53%), and *vgrG*3 (94.49%) with V. cholerae O1 El Tor strain N16961. In addition, thermolabile hemolysin (*tlh*) is also considered a signature molecular marker for the species (36). This gene is rarely reported from nonclinical strains. The DNA sequence of *tlh* identified in the strain PS-4 showed 60% similarity with Vibrio parahaemolyticus. Thus, the Vibrio cholerae strain from the pufferfish skin adds a new ecological niche to this bacterium.

**TABLE 2:** Virulence factors of Vibrio cholerae strain PS-4

Virulence factor	Virulence genes^*a*^		Locus position
	Chr. I	Chr. II	
Secretion system	–	Type VI secretion system protein (*vasA*)	44800–46569
	–	Type VI secretion system protein (*vasB*)	43820–44836
	–	Type VI secretion system protein (*vasC*)	42333–43817
	–	Type VI secretion system protein (*vasD*)	41854–42330
	–	Type VI secretion system protein (*vasE*)	40513–41847
	–	Type VI secretion system protein (*vasF*)	39737–40510
	–	Type VI secretion system protein (*clpB*/*vasG*)	37102–39711
	–	Type VI secretion system protein (*vasH*)	35507–37099
	–	Type VI secretion system protein (*vasI*)	34854–35537
	–	Type VI secretion system protein (*vasJ*)	33435–34844
	–	Type VI secretion system protein (*vasK*)	29874–33419
	–	Type VI secretion system protein (*vasL*)	28561–29826
	–	Type VI secretion system tubule-forming protein A (*vipA*)	48534–49040
	–	Type VI secretion system tubule-forming protein B (*vipB*)	47015–48493
	–	Type VI secretion system substrate Hcp-2 (*hcp-2*)	168375–168893
	–	Type VI secretion system protein (*VCA0109*)	46575–47012
	–	Type VI secretion system protein (*VCA0122*)	28268–28510
	–	Type VI secretion system substrate (*vgrG-2*)	166163–168247
	–	Type VI secretion system substrate (*vgrG-3*)	25333–28308
Toxin	–	Thermolabile hemolysin (*tlh*)	932592–932858
	–	Cytolysin VCC (*hlyA*)	927910–930135
	Type I secretion C-terminal target domain-containing protein (*rtx*)	–	1262235–1264250
Adherence	Aldehyde dehydrogenase (*aldA*)	–	1092438–1093958
	*N*-Acetylneuraminate lyase (*nanA*)	–	1107734–1108630
	–	Methyl-accepting chemotaxis protein (*VC0512*)	1974220–1976571

a –, not detected.

## MATERIALS AND METHODS

### Bacterial strain and growth medium.

The pufferfish (Tetraodon cutcutia) samples were collected from Mahanadi River, India (coordinates: 20°26′46.6″N 85°44′28.3″E), in August 2018 and transported to the laboratory in a plastic container with river water. Mucus on pufferfish skin was taken using sterile cotton swabs and transferred into 1 mL of sterile phosphate-buffered saline (PBS), pH 7.4, to isolate bacteria. The bacteria from the cotton swabs were suspended in PBS by vigorous vortexing. The suspension was used as a master mix (37) for the isolation of bacteria. An aliquot (100 μL) of master mix sample was serially diluted using PBS and plated onto nutrient agar (BD, Difco). All plates were incubated at 30°C corresponding to the river water temperature for 2 days. Several colonies developed at 30°C were picked and purified by repeated streaking on the same medium. Cultures were maintained on nutrient agar (BD, Difco) and stored at 4°C for short-term preservation. For long-term preservation, the culture was kept at −80°C in 15% (vol/vol) glycerol.

### Phenotypic features and serogroup identification of V. cholerae strain PS-4.

Gram staining was carried out using the commercial kit (Becton, Dickinson, USA). Oxidase activity was tested with discs impregnated with dimethyl *p*-phenylenediamine (Hi-Media, India). Catalase activity was performed by mixing a freshly centrifuged culture pellet with a drop of hydrogen peroxide (10% [vol/vol]). Growth and reaction to ferment sucrose were tested on TCBS agar medium (BD, Difco). Utilization of sugars was tested separately by adding 0.5% concentration of glucose or fructose in Luria-Bertani agar medium (BD, Difco) containing bromothymol blue (2.0 mg/L) as a pH indicator at 37°C for 48 h. To ascertain hemolytic activity, strain PS-4 was streaked on Columbia blood agar base supplemented with 5% (vol/vol) defibrinated sheep blood followed by incubation at 37°C for 48 h (37). Preparation of O antisera and slide agglutination were performed as previously described (38).

### Identification of bacteria by 16S rRNA sequencing.

Genomic DNA was extracted following the methods of Sambrook and Russel (39), and PCR was carried out using the universal bacterial primers 27F (5′-GAGTTTGATCCTGGCTCAG-3′) and 1525R (5′-AAAGGAGGTGATCCAGCC-3′) (40). The PCR product was purified using a QIAquick gel extraction kit (Qiagen) and sequenced in a capillary DNA analyzer (3500, Applied Biosystems) following the manufacturer’s protocol. The 16S rRNA gene sequences were assembled using the sequence alignment editor program BioEdit (41) and compared with those in GenBank after BLAST searches (42) and using the EzBioCloud Database (43).

### Whole-genome sequencing and annotation.

The genomic DNA of Vibrio cholerae strain PS-4 was isolated using standard methods by Sambrook and Russel (39). DNA concentration and quality were measured using a NanoDrop 8000 spectrophotometer (Thermo Scientific). A combination of both short-read Illumina and long-read Oxford Nanopore sequencing platforms was used to generate the high-quality complete genome sequence of V. cholerae strain PS-4. Illumina short-read DNA sequencing was carried out as described earlier (37). For long-read Nanopore sequencing, a genomic library was prepared using the Nanopore ligation sequencing kit (SQK-LSK109; Oxford Nanopore, Oxford, UK). The library was sequenced with an R9.4.1 MinION flow cell (FlO-MIN106) using MinKNOW v2.0 with the default settings. Barcode and adapter sequences from Nanopore long reads were trimmed using Porechop v0.2. (https://github.com/rrwick/Porechop), and reads with a minimum of 1 kb in length were filtered using seqtk v1.2 (https://github.com/lh3/seqtk) for downstream analysis. The hybrid genome assembly was performed using Unicycler version 0.4.9 (44) in hybrid assembly mode. The highly accurate Illumina short reads were aligned against the long Nanopore reads to sort out random sequencing errors (44). The assembled genomes were annotated using the NCBI Prokaryotic Genome Annotation Pipeline (PGAP; version 4.9) with default parameters (45). Completeness and contamination of the whole-genome sequence were measured using CheckM (46). Genomic G+C content and assembly statistics were determined using Perl script (https://github.com/tomdeman-bio/Sequence-scripts/blob/master/calc_N50_GC_genomesize.pl).

### Comparative genomics.

We used bioinformatics tools to compare the genomic relatedness of strain PS-4 with reference genomes of validly published 131 type strains of *Vibrio* available in the NCBI database (last accessed 25 March 2021). The advent of next-generation sequencing and bioinformatics tools made it possible to compare genomic data by *is*DDH, ANI, and AAI values. The ANI was calculated using the Python module pyani (https://github.com/widdowquinn/pyani) with the ANIb method. *In silico* DDH similarity was measured with the help of the genome-to-genome distance calculator (formula 3) (31). Average amino acid identity (AAI) was estimated using the “aai_wf” function implemented in the compareM program (https://github.com/dparks1134/CompareM).

### Genome-wide SNP determination and phylogenetic analysis.

For SNP-based phylogenetic analysis, 70 complete or draft genome sequences of V. cholerae strains were retrieved from the NCBI database. Single-nucleotide polymorphisms (SNPs) were identified from genome assemblies using V. cholerae strain N16961 as a reference for alignment using Snippy version v4.6.0 (https://github.com/tseemann/snippy). The recombinant region was removed using the default parameters of Gubbins version 2.3.4 (47). Core SNPs were extracted with the help of SNP sites (48), and a maximum-likelihood (ML) phylogenetic tree was constructed using RAxML version 8.2.4 (49) with GTRGAMMA model (50) for nucleotide substitution with gamma-distributed rate heterogeneity.

In addition, the use of whole-genome sequences has been regarded as a promising avenue to determine the phylogenetic position of microorganisms. Analysis of evolutionary phylogeny based on core genomes is the gold standard for strain identification, superior to those found on a single gene marker or concatenated sequences of a few genes. Therefore, we performed the phylogenomic analysis based on genome-wide core genes of the available whole-genomes of 131 type strains of all species with correct validly published names of *Vibrio* with more than 95% genome completeness. We retrieved the genome sequence of the type strains from the NCBI database (https://github.com/kblin/ncbi-genome-download/). The core genes were extracted by the up-to-date bacterial core gene (UBCG) pipeline (51). The genes were concatenated, and a maximum-likelihood tree was reconstructed with the genetic testing registry (GTR) model using the RAxML tool (52). Further, the nonrecombinant core genome-based phylogenetic tree was constructed following Mateo-Estrada et al. (53).

### Comparative analysis of virulence genes.

Virulence-associated proteins of strain PS-4 were identified using the blastp program against the virulence factor database (VFDB) (54) with the following parameters: identity cutoff of 75%, coverage cutoff of 70%, and E value cutoff of 1×10^−5^. The virulence-related genes of strain PS-4 were compared with the O1/O139 type of Vibrio cholerae and non-O1/non-O139 V. cholerae serogroup strains using the blastn algorithm (55). The heat map was generated from nucleotide percentage identity employing Manhattan distance and average clustering method using the heatmap2 function of the gplots package (56) in R (57).

### Data availability.

The GenBank/EMBL/DDBJ accession numbers for the genome and 16S rRNA gene sequences of Vibrio cholerae strain PS-4 are CP077197 (chromosome I), CP077198 (chromosome II), and MW926953, respectively.
